# The Role of BH3-Mimetic Drugs in the Treatment of Pediatric Hepatoblastoma

**DOI:** 10.3390/ijms16024190

**Published:** 2015-02-16

**Authors:** Justus Lieber, Sorin Armeanu-Ebinger, Jörg Fuchs

**Affiliations:** Department of Pediatric Surgery and Pediatric Urology, University Children’s Hospital, Hoppe-Seyler-Strasse 1, Tübingen D-72076, Germany; E-Mails: armeanu@uni-tuebingen.de (S.A.-E.); joerg.fuchs@med.uni-tuebingen.de (J.F.)

**Keywords:** pediatric hepatoblastoma, multi drug resistance, metastases, modulation of apoptosis, BH3-mimetic drugs, ABT-737, obatoclax

## Abstract

Pediatric hepatoblastoma (HB) is commonly treated by neoadjuvant chemotherapy and surgical tumor resection according to international multicenter trial protocols. Complete tumor resection is essential and survival rates up to 95% have now been achieved in those tumors classified as standard-risk HB. Drug resistance and occurrence of metastases remain the major challenges in the treatment of HB, especially in high-risk tumors. These conditions urgently require the development of alternative therapeutic strategies. One of those alternatives is the modulation of apoptosis in HB cells. HBs regularly overexpress anti-apoptotic proteins of the Bcl-family in comparison to healthy liver tissue. This fact may contribute to the development of chemoresistance of HB cells. Synthetic small inhibitory molecules with BH3-mimetic effects, such as ABT-737 and obatoclax, enhance the susceptibility of tumor cells to different cytotoxic drugs and thereby affect initiator proteins of the apoptosis cascade via the intrinsic pathway. Besides additive effects on HB cell viability when used in combination with cytotoxic drugs, BH3-mimetics also play a role in preventing metastasation by reducing adhesion and inhibiting cell migration abilities. Presumably, including additive BH3-mimetic drugs into existing therapeutic regimens in HB patients might allow dose reduction of established cytotoxic drugs and thereby associated immanent side effects, while maintaining the antitumor activity. Furthermore, reduction of tumor growth and inhibition of tumor cell dissemination may facilitate complete surgical tumor resection, which is mandatory in this tumor type resulting in improved survival rates in high-risk HB. Currently, there are phase I and phase II clinical trials in several cancer entities using this potential target. This paper reviews the available literature regarding the use of BH3-mimetic drugs as single agents or in combination with chemotherapy in various malignancies and focuses on results in HB cells.

## 1. Introduction

Hepatoblastoma (HB) is the most common primary malignant pediatric liver tumor in children with an incidence of 1.2 per million and an overall median age at diagnosis of 18 months [1]. Histologically, the tumors are divided into epithelial and mixed epithelial/mesenchymal subtypes. Tumor cells may appear with a wide variety of characteristics ranging from almost liver-cell-like to undifferentiated blastomal cells. The majority of HB is epithelial, consisting of embryonal and fetal cells. About 5% of the tumors belong to the small-cell undifferentiated subtype, which is associated with a worse prognosis [2]. HB usually expresses α-fetoprotein (AFP), which is also elevated in the serum of 90% of children with this tumor. AFP serves as a tumor marker, as an indicator for treatment response, and as a follow up marker to detect early relapses. For laboratory experiments, the HB cell lines HepT1 (derived from a multifocal embryonal HB [3]) and HuH6 (derived from a mixed HB with focal chondroosteogenic tissue and a squamous cell morphology [4]) were eligible.

## 2. Established Treatment Strategies against HB

To date, treatment strategies against HB have been established, constantly evaluated, and revised by cooperative study groups (Childhood Liver Tumor Strategy Group (SIOPEL), the Children’s Oncology Group (COG), and the national study groups from Germany (GPOH) and Japan (JPLT)) [5,6]. The main goal of treatment is complete surgical resection of the tumor, because this is essential for survival of the patient [7,8]. However, surgery alone can cure very few patients with HB, because more than half of them present with unresectable primary tumors or distant metastases. Evidence that HB is a chemosensitive tumor has led to a combined treatment regiment consisting of surgery and chemotherapy [9]. Neoadjuvant chemotherapy usually leads to tumor shrinkage and makes the tumor more solid, less prone to bleeding and more demarcated from the remaining healthy liver parenchyma, which consequently leads to increased rates of complete tumor resection [10]. Also, potentially existing micrometastases in the lungs are treated early. The exact treatment protocol depends on the tumor stage. Currently, staging of tumors is usually performed according to the PRETEXT (Pretreatment Extent of Disease) system, which is based on pretreatment imaging using ultrasound, computed tomography (CT) scans and/or magnetic resonance imaging (MRI) [11]. It describes the site and size of the tumor, vascular invasion, and distant spread. The staging system identifies four PRETEXT stages (I–IV), which reflect the number of sections of the liver that are involved by the tumor and describes the extent of the disease beyond the liver using letters as mentioned in Table 1. In principle, HB tumors are stratified into risk groups, which differ slightly between the different international study groups.

**Table 1:** International risk stratification [12]. Risk stratification of children with Hepatoblastoma (HB) reflecting the respective international treatment protocols, COG: Children’s Oncology Group; SIOPEL: International Childhood Liver Tumour Strategy Group; JPLT: Japanese Pediatric Liver Tumour Study Group; GPOH: German Society of Pediatric Oncology and Hematology; SCU: small cell undifferentiated; AFP: α-fetoprotein, V (tumor extends into the vena cava and/or all three hepatic veins), P (the main and/or both left and right branch/es of the portal vein are involved by the tumor), E (evidence of extrahepatic intra-abdominal disease), and M (distant metastases).

Risk Group	COG	SIOPEL	JPLT	GPOH
**Very low risk**	PRETEXT I or II, pure fetal histology and primary resection	–	–	–
**Low risk/ Standard risk**	PRETEXT I or II, any histology primary resection	PRETEXT I, II, III	PRETEXT I, II, III	PRETEXT I, II, III
**Intermediate risk**	PRETEXT II, III, IV unresectable at diagnosis V+, P+, E+ SCU	–	PRETEXT IV, any tumor with rupture, N1,P2,P2a,V3, And V3a multifocal	–
**High risk**	Any PRETEXT, M+, AFP < 100ng/mL	Any PRETEXT, V+, P+, E+, M+, SCU, AFP < 100 ng/mL, tumor rupture	Any PRETEXT, M1, N2, AFP < 100 ng/mL	Any PRETEXT, V+, P+, E+, M+, multifocal

Standard-risk (SR)-HB are defined as PRETEXT stage I, II or III tumors without metastases, vascular involvement or extrahepatic disease. High-risk (HR)-HBs are PRETEXT stage IV tumors and/or one or more of the following criteria are present: Extrahepatic diseases (usually lung metastases), low AFP-values (<100 ng/mL), and/or tumor rupture [13].

The use of chemotherapy is uncontroversial in HB and it is applied according to the risk groups. Most SR-HB show a good response to chemotherapy, in which cisplatin has proven to be sufficient even applied as monotherapy [14]. Historically, combinations of cytotoxic drugs were use, such as cisplatin and doxorubicin (SIOPEL-3SR), or additional ifosfamid (German HB-99 study), but results were not superior to cisplatin monotherapy. On the contrary, intensity of monochemotherapy with cisplatin could be reduced while maintaining good treatment results [6,15]. For HR-HB, strategies were developed comprising intensification and/or combination of cytostatic chemotherapeutics, such as cisplatin, doxorubicin, vincristine, fluorouracil, carboplatin, ifosfamide, etoposide, and others in the North American, German, and Japanese trials [5,16,17,18,19,20]. However, the prognosis of patients with tumors involving all four liver sections or with distant metastases still remains unsatisfactory. For example in the SIOPEL trials, the 5-year event-free survival (EFS) in these patients after preoperative PLADO (cisplatin and doxorubicin) and delayed surgery (SIOPEL-1) was 46% (PRETEXT-IV), or 28% (metastases), respectively [21]. In a pilot study (SIOPEL-2HR) and the following SIOPEL-3HR trial, carboplatin was added to the PLADO backbone and led to improved survival in patients with PRETEXT-IV tumors (3-year EFS 68%) or metastases (56%) [15]. SIOPEL-4 aimed to further intensify chemotherapy in HR-HB by adopting a dense weekly dose administration of cisplatin in combination with monthly doxorubicin and delayed radical surgery [22]. Complete resection was achieved in 85% of patients including liver transplants and the 3-years EFS rate was 76%.

Even though optimization and intensification of chemotherapy leads to improved treatment results, it cannot eradicate primary tumors alone [6,16]. Surgical strategies for tumor resection include anatomic liver resections and liver transplantation [23,24]. Atypical, nonanatomic, or wedge resections are associated with a worse outcome, for which the presence of unappreciated microscopic vascular invasion and the known role of hepatocyte growth factor (HGF) in stimulating post-resection liver regeneration and residual tumor cell proliferation might be possible explanations [16,25]. Anatomic liver resection is recommended as lobectomy or segmentectomy at diagnosis for PRETEXT I, as lobectomy or trisegmentectomy after neoadjuvant chemotherapy for PRETEXT II and III, and as liver transplant or extreme resection for any PRETEXT IV. Although primary liver transplantation has excellent results, there is an ongoing discussion regarding the optimal procedure for tumors that are very large or critically positioned and impinge on essential vascular structures, and for tumors that are multicentric and present in all four sectors of the liver before neoadjuvant therapy. However, rescue transplantation (transplantation after initial liver resection) is not as effective as primary liver transplantation.

## 3. Specific Treatment Strategies against HB

Even though standard treatment protocols for HB have constantly been optimized, unsatisfactory results are still observed in some patients, especially in those with HR-HB. The main reasons for poor outcome is chemoresistance, unresectability of tumors or recurrence of disease. Therefore, various alternative treatment options have been proposed (Table 2).

**Table 2:** Selective alternative treatment proposals for HR-HB.

Option	Substance	Pathway	Reference
Gene-directed therapy with prodrugs	5-fluorocytosine	Converting non-toxic drugs into antiproliferative drugs	[26]
Kinase inhibitors	sorafenib, rapamycin	–	[27,28]
Control of gene expression	Epigenetic modulators	DNA methylation and histone acetylation	[29]
Protein homeostasis	Proteasome inhibitors	Degradation of proteins	[30]
Modulation of apoptosis	TNF-α, TRAIL	Induction of apoptosis, signal transduction	[31]
	–	Downregulation of Bcl-2 using siRNA	[32]
Toxification	High dose acetaminophen with *N*-acetylcysteine	–	[33]
Immunotherapy	Allogeneic graft-versus-HB effect.	Hematopoietic stem cell transplantation	[34]
	Natural killer cell-mediated lysis of hepatoma cells	Antitumor immune responses	[35,36]
Oncolytic virotherapy	Modified Adeno and Sendai viruses	Cancer-specific replication of viruses	[37,38]

## 4. Resistance Mechanisms in HB Cells

A major challenge for cancer treatment remains the development of drug resistance, a mechanism by which tumor cells achieve insensitivity to external insult or internal damage. Most HBs are generally chemosensitive; however, 80% of HBs develop drug resistance after four cycles of chemotherapy [39]. Several mechanisms in cancer cells to evade death signals have been identified contributing to multi-drug-resistance (MDR). Reports exist in this regard on overexpression of MDR-associated genes (e.g., *P*-glycoprotein), increased DNA repair, alternations in target molecules (e.g., topoisomerasis II), overexpression of detoxifying enzymes (e.g., Glutathion-*S*-Transferase), and others [40,41,42,43]. MDR is also caused by gene deletion or mutation (e.g., p. 53 [44]), which leads to deregulation of apoptosis.

**Figure 1:**
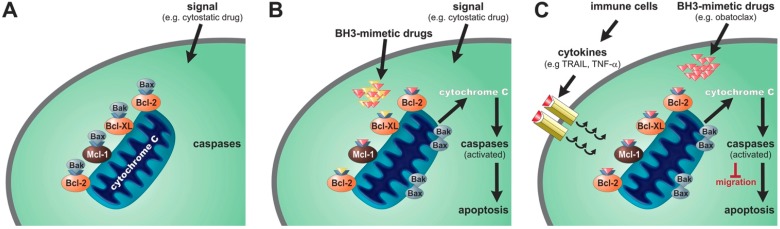
Effects of BH3-mimetic drugs. Overexpression of proteins of the Bcl-family in tumor cells cause capture of Bak and Bax in BH3-binding sites, which prevents initiation of the apoptosis cascade via intrinsic or extrinsic stimuli (**A**); BH3-mimetic drugs lead to the release of Bak and Bax, which oligomerize and insert as a complex in the outer mitochondrial membrane. This membrane permeabilisation is followed by cytochrome c release and other pro-apoptotic factors into the cytoplasm, initiating apoptosis and leading to cell death (**B**); BH3-mimetic drugs can enhance dead signals from immune cells (TRAIL, TNF-α) and can influence migration of tumor cells (**C**).

Apoptosis is an orchestrated cellular process important for maintaining homeostasis between cell proliferation and cell death, including the removal of diseased or damaged cells [45]. Apoptosis can be induced in two distinct pathways, both of which lead to the activation of effector caspases. The intrinsic pathway is substantially regulated by proteins of the B-cell lymphoma-2 (Bcl-2) family. Bcl-2 proteins share one of four Bcl-2 homology (BH1-4) domains, of which the BH3-domain is critical for mediating interactions among the family members [46]. Bcl-2 proteins can be grouped as anti-apoptotic (Bcl-2, Bcl-xL, Bcl-w, Mcl-1, and Bfl-1/A1) and pro-apoptotic. The latter group can be further divided as either multi-BH-domain proteins, including Bax and Bak, or as BH3-only proteins, such as Bid, Bad, Bim, Puma, and Noxa [47,48,49]. The BH3-domain is—in the presence of Bax and Bak—essential for the death function of BH3-only proteins [50]. Death signals caused by DNA-damage, deprivation of growth factors or activation of oncogenes lead to transcriptional or posttranslational modification of BH3-only proteins [49,51]. Consequently, Bak and Bax oligomerize and insert as complex in the outer mitochondrial membrane [50,52,53]. This membrane permeabilisation (MOMP = mitochondrial outer membrane permeabilization) is followed by cytochrome c release and other pro-apoptotic factors into the cytoplasm, initiating the apoptosis cascade and leading to cell death (Figure 1).

MOMP is regarded as one key element in the initiation of the apoptosis cascade [46]. A carefully regulated balance between pro-apoptotic and anti-apoptotic members of the Bcl-2 family determines the survival of the cell [54,55].

The extrinsic pathway of apoptosis is initiated on the plasma membrane upon ligation of death receptors and can be promoted by immune cells such as natural killer (NK) cells, T cells, and liver-specific Kupffer cells. These cells secrete proteins of the TNF (tumor necrosis factor) family such as TNF-α, FasL, and TRAIL (tumor necrosis factor-related apoptosis inducing ligand). Receptor binding recruits pro-caspase 8 into the death-inducing silencing complex. Cleavage of the BH3-only peptide, Bid, by caspase 8 links the apoptotic TRAIL signal to the mitochondrial pathway and the subsequent release of cytochrome c [56].

Overexpression of anti-apoptotic proteins or loss of pro-apoptotic members of the Bcl-2 family as well as a lost ability to express death receptors render cells correspondingly susceptible or resistant to apoptosis [57]. This mechanism is believed to contribute to tumor initiation and progression as well as to treatment resistance in various cancer types (colon, endometrial, Burkitt’s lymphoma, and ovary) [56,58,59]. Comparison of gene expression profiles of tumor samples with fetal liver tissue revealed downregulation of pro-apoptotic genes in HB samples as well, whereas a number of anti-apoptotic and prosurvival genes were upregulated [60,61]. In addition, TRAIL insensitivity has been described in HB cells, despite their expression of death receptors [62]. Therefore, modulation of apoptosis constitutes a promising therapeutic option for HB as it may resensitize tumor cells as targets of chemotherapeutic agents, mostly through induction of apoptosis.

## 5. Modulation of Apoptosis as a Concept of Anti-Tumor Therapy

The initial strategy of pharmacological inhibition of proteins of the Bcl-2 family was to use antisense oligonucleotides to inhibit transcription of mRNA in Bcl-2 [63]. Numerous other compounds with anti-cancer activity (e.g., epigallocatenin-3-gallate or chelerythrine) have been shown to antagonize the Bcl-2 family proteins. However, the clinical effect was only moderate [64,65]. Consequently, antagonization of function rather than reduction of proteins had been favored and small-molecule-inhibitors selective for anti-apoptotic Bcl-2 proteins have been identified [66,67]. Advances in crystallizing the Bcl-xL structure containing hydrophobic binding sites BH1–BH3, which usually bind to the α-helix of BH3-only proteins, has enabled the identification and synthesis of such small-molecular antagonists. They are in a competitive relationship with anti-apoptotic proteins and prevent the sequestration of pro-apoptotic BH3-only proteins such as tBid, Bad, Bax, and Bim [68]. Numerous natural organic compounds were found with BH3-mimetic effects of a broad affinity and specificity to Bcl proteins [46]. Currently, four Bcl-2 inhibitors are undergoing clinical trials: ABT-263 (navitoclax, an orally bioavailable analogue of ABT-737 [69], AT-101 [(−)-gossypol] [70], obatoclax (GX15-070) [71], and ABT-199 [72,73].

## 6. BH3-Mimetic Drugs as Sensitizers of Chemotherapy in HB

Small synthetic molecules with BH3-mimetic effects have been shown to enhance the intrinsic apoptotic pathway in several tumor cell lines. ABT-737 as single agent has shown moderate activity against several hematopoietic cell lines (leukemias, multiple myeloma, and cultured lymphoma) and some solid tumor cell lines, including prostate cancer, ovarian cancer, glioblastoma, and retinoblastoma. A high efficiency of single ABT-737 was only observed in small cell lung carcinoma [63,69,74,75,76,77,78,79,80]. In a xenograft model the highest efficiency of ABT-737 and a related compound, ABT-263, was observed when the IC_50_ *in vitro* was in a nanomolar range. Synergistic effects have been described with dexamethasone and melphalan in multiple myeloma and with cytotoxic drugs (e.g., paclitaxel, cisplatin, etoposide, doxorubicin) in a variety of tumor cell lines [67,81]. Obatoclax has also been shown to potentiate other cancer treatment approaches in xenograft models of small cell lung cancer, thyroid cancer, and colorectal cancer [70,82,83].

In HB cells, ABT-737 was found to induce apoptosis as a pan-Bcl-2 inhibitor at concentrations above 1 µM, whereas obatoclax similarly antagonized all anti-apoptotic Bcl-2 family proteins, including the dominant proteins Mcl-1 and Bfl-1, showing anti-apoptotic effects at a concentration as low as 0.03 µM [65,84]. Inhibition of these proteins using ABT-737 or obatoclax has induced significant reduction of HB cell proliferation [61,85]. It has also been demonstrated that these modulators of apoptosis enhance the effects of cytotoxic drugs *in vitro* and *in vivo*, where reduced proliferation rates were documented after combined treatment with ABT-737 and paclitaxel or cisplatin and reduction of tumor growth in a subcutaneous model of HB [86,87]. Other small molecular drugs with BH3-mimetic effect tested on HB cells, such as HA14-1 or TW37, did not show any significant effect as single agents, or in combination with several cytotoxic drugs [85].

ABT-737 inhibits the prosurvival function of Bcl-2, Bcl-xL, and Bcl-w, but exhibits low affinity to the anti-apoptotic Mcl-1 and A1 proteins. This anti-apoptotic group of Bcl-2 family proteins is frequently found to be overexpressed in numerous cancers including HB. Mcl-1 is expressed at high levels in HB, which are however inferior to expression levels in hepatocytes. This fact represents a relevant constraint for the efficiency of ABT-737. HB cells also express pro-apoptotic Bak, which has been described as key molecule for sensitizing tumor cells to ABT-737 [88,89]. However, the single-agent activity of ABT-737 is poor below doses of 1 mM. On the other hand it significantly potentiates the efficacy of established chemotherapeutic drugs on HB cells. Obatoclax has shown dose-dependent single-agent activity against HB cells at concentrations above 0.3 mM. Mechanistically, apoptosis induction by obatoclax can be preceded by liberation of Bak from Mcl-1, dissociation of Bim from Bcl-2, and Mcl-1 [90]. The additional binding on Mcl-1 proteins may enhance efficiency of obatoclax; however, gene expression analysis revealed a two-fold lower expression of Mcl-1 in native HB tissue and HuH6 cells than in normal liver tissue and a benefit of obatoclax was not expected [91,92]. On the other side, it has been proposed that obatoclax abolishes cell growth independently of apoptosis by inducing a S–G2 cell cycle block suggesting multiple targets of this agent [77]. These Bcl-2 independent targets of obatoclax may have clinical applicability, but mechanisms of these anti-proliferative effects on HB cells in particular require further investigations.

ABT-737 and obatoclax also enhance cytotoxic effects when combined with cisplatin, doxorubicin, etoposide, and paclitaxel, which are commonly used in treatment protocols of HB [6,93]. Cisplatin is the most important cytotoxic drug in the treatment of HB, and leads to an excellent 3-year survival rate of 96% in SR-HB, even when applied as monotherapy [14,21]. Therapy has been intensified in HR-HB using cisplatin monotherapy and second-line cytostatic drugs. However, significant irreversible adverse effects have been observed, such as nephro- and neurotoxicity as well as myelodepression or heart failure. Therefore, BH3-mimetic substances seem promising since they might enable dose reduction of cytotoxic drugs while maintaining their antitumor activity.

In general, the effects of ABT-737 and obatoclax were more relevant in HuH6 cells than in HepT1 cells. Higher concentrations of ABT-737 and obatoclax were used in HepT1 cells, but viability was reduced in HuH6 only. This enhanced sensibility of HuH6 cells does not correlate with the relative expression of anti- and pro-apoptotic proteins, as Bcl-xL, Bax, and Mcl-1 are similarly expressed in both cell lines. Therefore, we assume that a higher proliferation rate in HuH6 cells may explain the higher sensibility.

HA14-1 is an organic compound originally discovered by computer modeling. It is the first small molecule, which was predicted to bind to Bcl-2 with inhibitory effects [94]. HA14-1 has been shown to induce apoptosis in various hematopoietic and solid tumor cell lines, such as leukemias, lymphomas, breast and ovarian carcinomas, malignant glioma, multiple myeloma, and neuroblastoma [71,83,95,96,97,98]. A synergism with a variety of anti-cancer agents has also been described [99,100]. In HB cells no pro-apoptotic effects could be observed with HA14-1. A variable response ranging from partial to massive cell death has been described in other cell lines and it is known, that expression of HA14-1 targets (Bcl-2 and Bcl-xL) did not correlate to these different responses; consequently, the potentiating effect of HA14-1 might be drug- and cell-type specific [75]. HA14-1 is also highly unstable and may rapidly decompose to inactive compounds. Decomposition generates reactive oxygen species (ROS) resulting in potent pro-apoptotic activity, which makes interpretation of results in addition to effects of antagonizing anti-apoptotic Bcl-2 family proteins difficult [101,102]. In summary, HA14-1 did not show effects on HB cells in general and did not enhance effects when combined with cytotoxic drugs [85].

The anti-tumor action of TW-37; which also binds to the BH3 groove of Bcl-2; is assumed to be due to a combination of a pro-apoptotic and specific anti-angiogenic effects as described in head and neck small cell carcinoma; pancreatic cancer; and lymphoma cells. This effect has been observed as single agent and in combination with cytotoxic drugs [76,103]. In HB cells; effects of TW-37 were moderate. Surprisingly; no additive effects after combination with cisplatin or other tested cytotoxic drugs were seen; as reported for head and neck squamous cell carcinomas (HNSCC); for which cisplatin is also commonly used in current treatment protocols [104]. Both; HNSCC and HB; express high levels of Bcl-2; so that the mechanism for the absence of additive effects in HB cells remains unclear. The IC_50_ for cisplatin in HuH6 and HNSCC (UM-SCC-1; UM-SCC-74A) was comparable; but required doses of TW-37 to significantly enhance effects of the combined treatment were three-fold lower in HNSCC.

## 7. BH3-Mimetic Drugs as Sensitizers of the Immune System in HB

The extrinsic pathway of apoptosis can be promoted by various immune cells that express proteins of the TNF family, such as TRAIL and/or TNF-α [105]. Thus, clinical trials in cancer patients have utilized soluble recombinant TRAIL and agonistic monoclonal antibodies that target TRAIL receptors as well as TNF-α. However, its clinical use is limited by severe dose-limiting toxicity [31,106]. In addition, liver tumors exhibit a variety of TRAIL and TNF-α resistance mechanisms, which emphasizes the necessity of a mechanism to restore the sensitivity of the tumor cells to receptor-mediated cytotoxicity [62]. TRAIL significantly induces apoptosis in both HB cell lines when combined with low concentrations of obatoclax [107]. This effect was moderate with ABT-737, but may be the result of the Mcl-1 expression in HB cells, which can be targeted more effectively using obatoclax. Induction of apoptosis was also significantly enhanced when HuH6 cells were treated with obatoclax in combination with TNF-α. These findings suggest that rather than local administration of tumor necrosis proteins, a broad modulation of apoptotic molecules using BH3-mimetic drugs can be used to sensitize transformed liver cells to TNF-α or TRAIL as previously observed with proteasome inhibitors such as bortezomib or histone deacetylase inhibitors [62,108]. Moreover, TRAIL is critically involved in tumor rejection through cell-mediated immune surveillance. Various immune cells, such as NK cells, T cells, and liver-specific Kupffer cells express TRAIL and mediate apoptosis in infected or transformed cells [109]. In addition, increased expression of stress molecules in tumor cells, such as MICA/B, can be initiated through activation of NK cells by BH3-mimetic drugs. This is then followed by effective tumor cell lysis [35,62]. HB dissemination in the liver was impaired in a mouse model as a result of the interaction of Kupffer cells with HB cells in the presence of BH3-mimetic drugs [107].

## 8. BH3-Mimetic Drugs as Inhibitors of Tumor Cell Migration

The capacity to migrate and to invade foreign tissues is a common feature of cancer cells dramatically contributing to the malignancy of the disease. Development of metastases in HB also remains a major challenge in treatment. Dissemination and metastasation is closely linked to cell adhesion and cell migration ability, which is also influenced by Bcl-2 proteins [110]. In Bcl-2^−/−^ cells, knockdown of Bcl-2 reduced the cell adhesion of extracellular matrix proteins (ECM). Moreover, knockdown of Bcl-2 impaired the adhesion of ureteric bud cells to vitronectin and fibronectin [111]. In colorectal cancer cells downregulation of Mcl-1, Bcl-xL or Bcl-2 could be demonstrated to lead to a striking impairment of migration and invasion [112]. This is in line with the observation that HB cells adhere less efficiently to matrigel in the presence of ABT-737 or obatoclax, blocking the anti-apoptotic proteins. Similar observations were made with hepatocellular carcinoma cells [113]. Reduced adhesion to ECM may promote or reduce occurence of metastasation depending on the most important events responsible for metastasation in solid tumor cells: detachment from the tumor mass and anchorage of circulating tumor cells to distant tissues. At present, there exist no models to study spontaneous metastasation of HB. However, it could be shown that obatoclax inhibits invasion of HB in the liver in an orthotopic model of HB [107]. In this context, BH3-mimetic drugs are discussed to impair development of lamellipodia in HB cells. Caspases enhance degradation of the GTPase Cdc42, which belongs to the Rho-family and regulates signaling pathways that control diverse cellular functions including cell morphology, migration, endocytosis and cell cycle progression. Other components of the tumor cytoskeleton such as actin, gelsolin, lamin, and plectin are also targets of caspases during early apoptotic events. Moreover, key enzymes, that organize the F-actin network with GTPase activity, are direct substrates of caspase-3 and -7, and play an important role in linking apoptosis to cell motility. BH3-mimetics disrupt the interaction of anti-apoptotic proteins Bcl-2 and Bcl-xL with Beclin-1. Subsequently, the vacant Beclin-1 can trigger the autophagy pathway. However, this has not been studied so far in HB cells.

## 9. Therapeutic Contribution of BH3-Mimetic Drugs in HB

Survival rates of children with HB depend on prognostic factors such as histological classification, stage, resectability, presence of metastases, AFP levels, patients’ age, and molecular-genetic markers [114,115]. In principle, risk stratification of patients has been improved and has led to encouraging treatment regimens and improved late results [22]. Nevertheless, a group within HR-HB exists, which urgently requires the development of alternative therapeutic strategies. The SIOPEL has modified the risk stratification using the data of more than 1600 patients from international trials collected in a common database CHIC (Children’s Hepatic Tumor International Collaboration) to establish a new international treatment protocol including alternative regiments. In this context, measurement of apoptosis-relevant expression of proteins in patients with HB appears reasonable. Inhibition of Bcl-2 molecules using BH3-mimetic drugs has shown a significant reduction of cell proliferation and tumor growth. This concept has also proven to sensitize immune cells to tumor cells and to inhibit tumor cell dissemination and migration. Consequently, this specific mechanism to restore effectors of apoptosis initiation represents a broad and high level of importance and therefore should be further evaluated in preclinical models as an option for selected HR-HB, especially in those patients presenting with an increased expression of anti-apoptotic proteins.

Currently, obatoclax is under investigation in several clinical trials including those targeting malignant solid tumors. It has been described to be well tolerated without dose-limiting toxicity at 28 mg/m^2^, although some minor CNS-related side effects have been observed [77,81,116,117]. In addition, a combination therapy using obatoclax together with platin derivates or etoposide has been applied in phase II studies of small cell lung cancer even though some transient side effects have been described in some patients [118]. However, the safety profile of obatoclax makes it an attractive candidate for inclusion in other solid tumor malignancies including childhood HB.

Recently, re-engineering of ABT-263 (navitoclax), creating ABT-199, has been reported by Souers and colleagues [119]. ABT-199 maintains a sub-nanomolar affinity for Bcl-2, but binds at a very significantly less effective level to Bcl-xL. This suggests that the drug may not cause clinically significant thrombocytopenia as described for obatoclax and ABT-263 [117,120]. Suppression of tumor growth by ABT-199 has been described in several human hematological tumor xenograft models with an additive efficacy in combination with traditional cytotoxic agents [121,122,123]. On the other side, tumor lysis after treatment with ABT-199 has been observed to such an extent that potentially serious complications occur. This will have to be critically taken into consideration during a further clinical assessment of this orally bioavailable selective Bcl-2 inhibitor [124].

Including additive BH3-mimetic drugs within existing treatment regimens of HB may lead to dose reduction of traditional cytotoxic drugs and to a reduction of associated immanent side effects, such as nephrotoxicity, ototoxicity and cardiotoxicity while maintaining the antitumor activity at the same time. Furthermore, reduction of tumor growth and inhibition of tumor cell dissemination may presumably facilitate complete surgical tumor resection and improves outcome of HR-HB. Clinical studies should address activation of the immune system in the context of BH3-mimetic drugs as suggested by results of several studies showing various mechanisms of BH3-induced tumor cell death.

## 10. Conclusions

Treatment optimization through international multicenter trials of HB improved results in SR tumors. In contrast, drug resistance and occurrence of metastases remain the major challenges in the treatment of HR-HB, which urgently requires the development of alternative therapeutic strategies. BH3-mimetic drugs represent a new and promising class of agents in cancer treatment, affecting not only apoptosis modulation but also the immune response and metastases. Convincing preclinical data suggest BH3-mimetics for a clinical trial in pediatric HB.
